# Modulation of BRD4 in HIV epigenetic regulation: implications for finding an HIV cure

**DOI:** 10.1186/s12977-020-00547-9

**Published:** 2021-01-07

**Authors:** Edrous Alamer, Chaojie Zhong, Renee Hajnik, Lynn Soong, Haitao Hu

**Affiliations:** 1 Department of Microbiology and Immunology, University of Texas Medical Branch (UTMB), MRB 4.142A, 301 University Blvd, Galveston, TX 77555 USA; 2 Department of Medical Laboratories Technology, College of Applied Medical Sciences, Jazan University, Jazan, Saudi Arabia; 3 Medical Research Center, Jazan University, Jazan, 45142 Saudi Arabia; 4 Institute for Human Infections and Immunity, Sealy Institute for Vaccine Sciences, University of Texas Medical Branch, Galveston, TX 77555 USA

**Keywords:** BRD4, Epigenetic regulation, HIV, Latency

## Abstract

Following reverse transcription, HIV viral DNA is integrated into host cell genomes and establishes a stable latent infection, which has posed a major obstacle for obtaining a cure for HIV. HIV proviral transcription is regulated in cellular reservoirs by complex host epigenetic and transcriptional machineries. The Bromodomain (BD) and Extra-Terminal Domain (ET) protein, BRD4, is an important epigenetic reader that interacts with acetyl-histones and a variety of chromatin and transcriptional regulators to control gene expression, including HIV. Modulation of BRD4 by a pan BET inhibitor (JQ1) has been shown to activate HIV transcription. Recent studies by my group and others indicate that the function of BRD4 is versatile and its effects on HIV transcription may depend on the partner proteins or pathways engaged by BRD4. Our studies have reported a novel class of small-molecule modulators that are distinct from JQ1 but induce HIV transcriptional suppression through BRD4. Herein, we reviewed recent research on the modulation of BRD4 in HIV epigenetic regulation and discussed their potential implications for finding an HIV cure.

## Introduction

Human immunodeficiency virus (HIV) continues to cause a global pandemic with nearly 38 million people infected worldwide. While antiretroviral therapy (ART) effectively suppresses active HIV replication and reduces plasma virus loads below detection limits (≤ 50 copies/ml) [1], it has significant limitations. ART fails to completely eradicate the virus, which is primarily because HIV establishes stable persistent and latent reservoirs (reviewed in [2–4]). ART withdrawal almost inevitably results in viral relapse [5–7], which occurs approximately 2–3 weeks after ART interruption [8]. Residual HIV expression is believed to be the cause of local inflammation and HIV-associated complications that are not reduced by ART intensification [9, 10]. HIV transcription from integrated viral genomes and viral particle release from stable cellular reservoirs are not affected by current anti-HIV drugs [11]. Therefore, new treatment paradigms beyond ART that are aimed at eradicating or curing HIV, have become a focus of current research. Following integration into the host cell genome, HIV provirus expression is regulated by a range of host epigenetic and transcriptional machineries (reviewed in [12–14]). In recent years, a variety of approaches directed towards these host mechanisms to induce HIV transcriptional activation (latency reversal) or suppression (deeper latency) have been actively pursued as potential HIV cure strategies.

## BRD4 and HIV epigenetic regulation

**Fig. 1:**
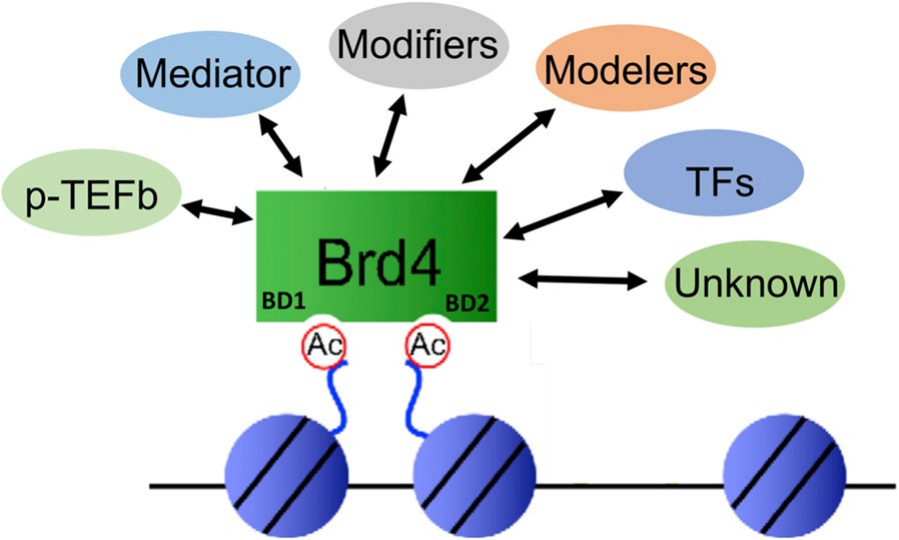
BRD4 protein–protein interaction (PPI) profile. Via the acetyl-lysine (KAc) binding pocket in BDs, BRD4 binds to acetylated-histones in chromatin and serves as a scaffolding platform for recruiting a spectrum of partner proteins through the PPI via domains such as ET, CTD (c-terminal domain), and PID (p-TEF-interacting domain). BRD4-interacting proteins include mediator, transcription factor, p-TEFb/CDK9, chromatin modifiers and modelers, and likely those not yet identified

BRD4 is a member of the bromodomain (BD) and extra-terminal (ET) domain family of proteins (BET). BET proteins consist of two conserved BDs (BD1 and BD2) that selectively bind to acetyl-lysine (KAc) residues in histones of chromatin [15, 16] and an ET domain that is involved in protein–protein interactions (PPI) [17]. As an epigenetic reader, BRD4 serves as a scaffolding platform through Ac-histone binding and interacts with many different chromatin and transcriptional regulators, such as p-TEFb/CDK9 [18–20], mediators [21], transcription factors (NF-kB) [22], and chromatin modifying and remodeling proteins [23], to regulate gene expression especially for primary response genes [24]. Accumulating evidence has indicated that the protein–protein interaction (PPI) network of BRD4 is complex and critical for the functional activity of BRD4 on target gene regulation (summarized in Fig. 1). Of relevance, targeted inhibition of BRD4 by small molecules, for example JQ1, can modulate its histone binding as well as dysregulate its PPI profile [25], which will be discussed subsequently. In addition to Ac-histone binding and PPI, BRD4 has been shown to possess some intrinsic kinase [26], histone chaperone [27], and acetyl-transferase [28] activities, supporting the idea that BRD4 is functionally versatile.

Since 2005, multiple studies have suggested that BRD4 plays a role in the regulation of HIV transcription and latency, although distinct results were reported by these studies [19, 23, 25, 29]. Early studies using retroviral LTR-driven reporter genes showed that BRD4 promotes HIV transcription and reverses latency [19]. More recent studies, however, indicated that BRD4 suppresses HIV transcription and promotes HIV latency [18, 23, 29]. In support of these later studies, inhibition of BRD4/BET by the pan-BET inhibitor JQ1 has been shown to activate HIV transcription [23, 25, 29]. Via its BDs, BRD4 can be recruited to the HIV promoter through binding to different acetyl-histones, including AcH3 and AcH4. It was more recently recognized that differential interactions of BRD4 with AcH4 vs. AcH3 leads to distinct effects on HIV transcriptional regulation [30]. Together, these early and recent studies further support the versatility of BRD4 and that its impact on HIV transcription and latency may depend on the partner proteins or pathways it engages. Therefore, a better understanding of the biology of BRD4 and how modulation of this epigenetic pathway could regulate HIV transcription/latency is needed.

## Modulate BRD4 by pan-BET inhibitors (BETi) to activate HIV transcription

Several small-molecule compounds have been developed to modulate human BET/BRD4. Some of them have shown promising safety profiles and have advanced to clinical trials for the treatment of human diseases [31, 32]. JQ1 is a cell-permeable small molecule developed by J. Bradner and colleagues in 2010 that competitively binds to acetyl-lysine (KAc) recognition motifs or the KAc binding site of bromodomains (BD) [33]. As a pan-BETi, JQ1 non-selectively binds to both BD1 and BD2 of BET proteins [33]. Interestingly, ensuing studies on the activity of JQ1 on HIV transcription revealed that JQ1 could activate HIV transcription and reverse latency in multiple cell line models [23, 25, 29, 34], although its effect on HIV transcription in primary CD4 T cells appears to be less remarkable [35]. In addition, JQ1 manifests synergistic effects in inducing HIV transcriptional activation in combination with other HIV latency reversing agents [36, 37].

An established function of BRD4 is regulating gene transcription elongation by recruiting the cellular super elongation complex (SEC) (e.g. p-TEFb/CDK9) to target gene promoters, stimulating RNA Pol II (RNAPII) activation and transcription elongation [19, 20]. As HIV integrates into host cell genomes, transcription of the integrated provirus requires host epigenetic and transcriptional machineries, such as RNAP-II and p-TEFb/CDK9. Mechanistic investigation indicated that modulation of BET/BRD4 by JQ1 relieves the competition between BRD4 and HIV Tat for cellular CDK9, facilitating the binding of CDK9 to Tat and thereby enhancing Tat-mediated HIV transcription elongation [25]. In this case, the activity of JQ1 is believed to be Tat dependent as JQ1 activates HIV LTR more potently in cells expressing Tat [25]. However, other studies reported similar latency-reversing activities for JQ1 in A72 J-Lat cells (which contain a latent LTR-GFP construct lacking Tat), indicating the existence of Tat-independent mechanisms for JQ1 as well [38]. In support of this, it was more recently shown that JQ1 induces dissociation of BRD4 from the repressive chromatin remodeling proteins (SWI/SNF) at the HIV LTR, thereby reversing BRD4-mediated HIV transcriptional suppression [23]. This is considered to be independent of Tat-mediated transcription elongation but dependent on the regulating chromatin structure at the HIV LTR.

In addition to JQ1, several other BET inhibitors have been tested in HIV infection models with some compounds demonstrating HIV latency-reversing activities, such as UMB-136 [39], MMQO [40], OTX015 [41], and I-BET151[42]. Mechanistically, OTX015 increases the occupancy of CDK9 at the HIV-1 LTR and activates RNAP II CTD phosphorylation, hence reactivating HIV expression [41]. MMQO mimics the acetylated lysines of core histones and interacts with the BET family protein BRD4. MMQO reactivates HIV expression independently of Tat through an unknown mechanism [40]. Together, the majority of these BET inhibitors (including JQ1) have shared mechanistic properties, including lack of selectivity for different BET proteins and primarily targeting the acetyl-lysine (KAc) recognition site of BET bromodomains (BD) for binding and modulation.

## Modulation of BRD4 by ZL0580 to induce HIV epigenetic suppression

Through structure-aided design, our recent studies [43, 44] tested a novel class of small molecules that were designed to selectively target BRD4 for their activities on HIV transcription. Intriguingly, our studies have identified a lead small molecule (ZL0580) and several analogs that are distinct from JQ1, but which induce HIV transcriptional suppression via BRD4. The suppressive activity of ZL0580 was confirmed for both transcriptionally active and latent HIV in multiple cell models, including J-Lat cells, human PBMCs/primary CD4 T cells [43], and human myeloid cells/microglia [44]. Using time-resolved fluorescence energy transfer (TR-FRET) assay, our study identified that unlike JQ1, which is a pan-BET inhibitor that binds to both BD1 and BD2 of all 4 BET proteins [33], ZL0580 selectively binds to BD1 of BRD4 over other BET proteins [43]. Our functional analyses (BRD4 knockout and overexpression studies) verified that the suppressive effect of ZL0580 on HIV is mediated by BRD4 [43]. Our recent studies therefore support the notion that human BRD4 and its associated epigenetic machinery can be modulated to repress HIV transcription.

### ZL0580 inhibits HIV transcription in J-Lat cells and human PBMCs/CD4 T cells

Our study [43] initially screened this novel class of BRD4 modulators for their activities on HIV transcription in J-Lat cells (full-length; 10.6) and identified ZL0580 as a lead compound that induces suppression of both PMA-induced and basal HIV transcription in a dose-dependent manner. The suppression was fairly durable with a single dose leading to significant transcriptional suppression for greater than 14 days in J-Lat cells. Notably, a more durable effect was also observed in microglial cells [44] (to be discussed below). This durable effect could be due to the induction of a repressive chromatin structure at the HIV LTR by ZL0580 [43], which is considered critical for the utility of this class of molecules as a potential “block and lock” HIV therapeutic approach. Another key observation further supporting the epigenetic effect of our compound on HIV is that pretreatment of J-Lat cells with ZL0580 renders them resistant to latent HIV reactivation by latency-reversing agents (SAHA and Prostratin). Other than J-Lat cells, this compound was also tested in human CD4 T cells that were infected with HIV in vitro, which is considered a more physiologically relevant system. ZL0580 could suppress HIV even more potently in CD4 T cells (EC50 =  ~ 2.5 µM) than in J-Lat cells (EC50 =  ~ 8uM). Similar to J-Lat, ZL0580 could also suppress HIV expression in both activated and resting human CD4 T cells. Analysis of T-cell phenotypes and activation markers (e.g. receptors, cytokines, chemokines, transcription factors, lineage differentiation factors, and innate restriction factors) indicated that there is no significant induction of a global impact on T cells by ZL0580 [43].

ART is effective in suppressing active HIV replication. However, low level viremia remains during ART treatment [45–47], and importantly HIV rebound can occur quickly after ART interruption [8, 48]. HIV blips may contribute to the replenishment of HIV reservoirs even under optimal ART therapy [49, 50] [51, 52], maximizing the potential emergence of drug-resistant strains. Our study [43] also explored synergistic effects of ZL0580 in combination with ART on repressing latent HIV using ex vivo PBMC samples from ART-treated, aviremic HIV-infected individuals [53, 54] and showed that our compound ZL0580 induces deep HIV latency by promoting HIV suppression during ART treatment and significantly delays viral rebound after ART cessation [43].

### ZL0580 inhibits HIV transcription in human myeloid/microglial cells

Myeloid cells and microglia are considered important HIV reservoirs in vivo and play a role in the establishment, pathogenesis and persistence of HIV, especially in the central nervous system (CNS). Relative to T cells, these cells have a longer lifespan [55, 56], are more resistant to cytopathic effects and are less sensitive to some ART drugs [57]. ART drugs have low efficiency in the CNS, which is partially due to their reduced ability to penetrate across the blood–brain barrier (BBB) [58, 59]. Therefore, even under ART and when peripheral HIV is suppressed, low levels of persistent HIV replication remain detectable in the CNS [60]. Our recent study [44] further expanded our findings on ZL0580 to human myeloid cells and microglia. The data showed that ZL0580 could also potently and durably suppress HIV in multiple myeloid cell lines (U1 and OM10.1) as well as in microglia (HC69) [61]. A single dose of ZL0580 induces suppression of basal HIV transcription in microglia through day 21, which was further prolonged to day 41 with two additional doses of ZL0580 (on day 3 and 7). Consistent with the results in J-Lat cells, pretreatment of microglia with ZL0580 renders them more resistant to latent HIV reactivation by latency reversal agents (LRAs). Our study further tested the anti-HIV activity of ZL0580 in human primary macrophages [monocyte-derived macrophage (MDM) infected with HIV in vitro] and demonstrated that ZL0580 promotes HIV suppression during ART treatment and prevented/delayed HIV rebound after treatment cessation [44]. Together, our recent studies [43, 44] provide evidence supporting the potential utility of our new BRD4 modulator ZL0580 as a “block and lock” HIV therapeutic approach. Based on these findings, our ongoing studies have aimed at chemically optimizing the lead molecule and identifying additional analogs that can induce HIV transcriptional suppression at higher potency but lower toxicity.

## Modulation of BRD4 by ZL0580 to suppress HIV: mechanisms of action

### Inhibition of Tat transactivation and HIV transcription elongation

HIV Tat protein is produced early after HIV transcription and plays a critical role in HIV transcriptional regulation [62]. Tat binds to the transactivation-responsive element (TAR) at the 5′ ends of nascent viral transcripts and recruits the cellular transcription elongation complex (including P-TEFb/CDK9) to the HIV promoter region. CDK9 can then stimulate RNA Pol II (RNAPII) activation and transcription elongation [63]. In the absence of Tat, HIV transcription elongation is aborted due to the activity of several negative factors [63]. Inhibition of Tat transactivation or disruption of Tat-CDK9 interaction represents an effective strategy for suppressing productive HIV transcription. Several strategies including synthetic, computational, and structural design methods have been attempted to identify Tat-TAR inhibitors [64]. For instance, TR87, a small TAR binder, was shown to suppress HIV replication in cell culture over 24 days [64]. Several Tat inhibitors are reported with evident suppressive effects on HIV transcription [11]. Indeed, recent studies have shown that Tat inhibition by dCA reduces residual viremia during ART treatment and prevents viral rebound after ART interruption in virally suppressed, HIV-infected humanized mice [65]. However, potential viral resistance still exists when targeting Tat protein. HIV has a significant ability to escape selective pressure [66–68], and resistance to antiretroviral drugs is commonly seen, particularly when used as monotherapy [69, 70].

**Fig. 2:**
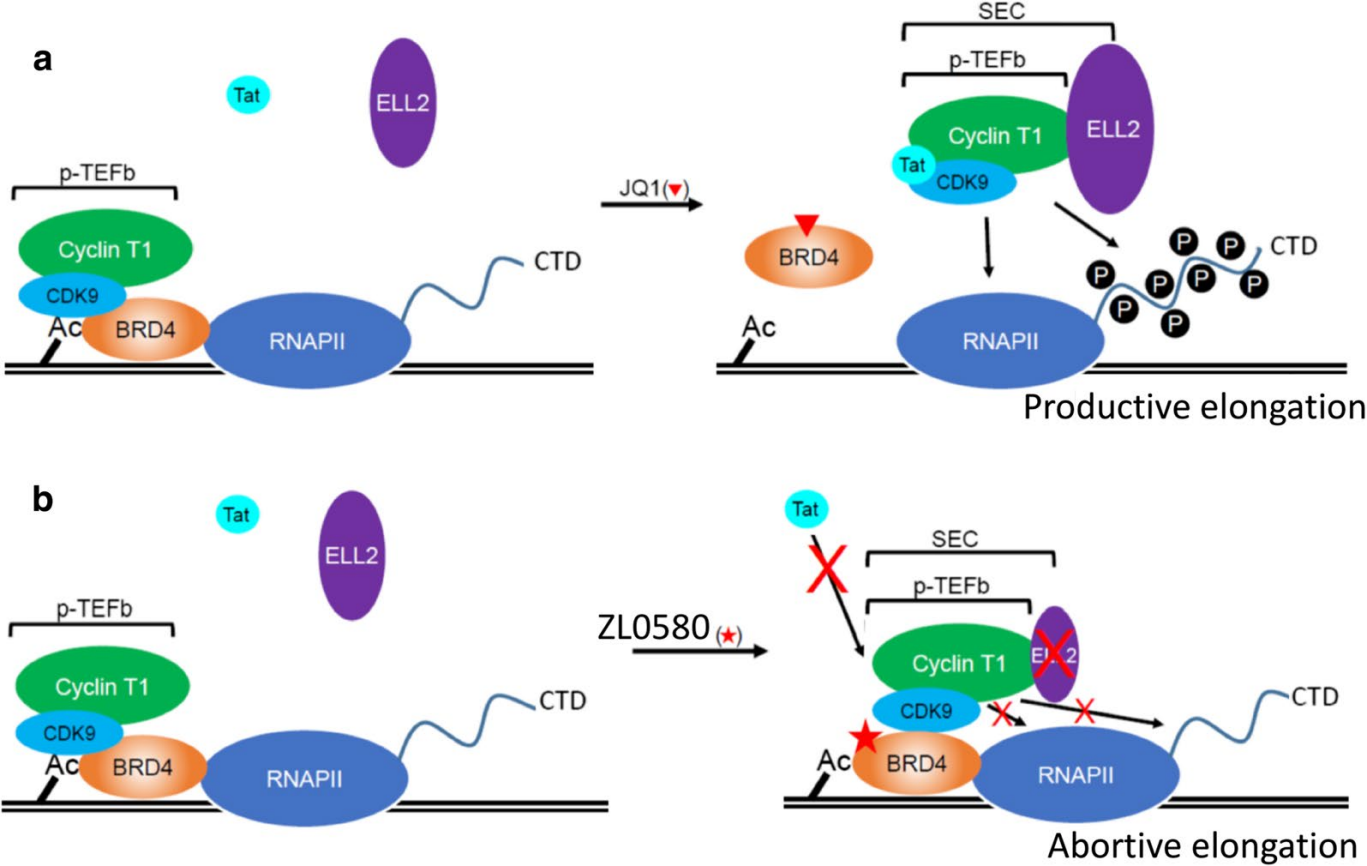
A proposed model for the distinct effects of ZL0580 and JQ1 on BRD4 PPI and Tat-mediated HIV transcription elongation. **a** BRD4 inhibition by JQ1. In the absence of JQ1 (left), BRD4 competes with HIV Tat for cellular p-TEFb/CDK9 and thereby suppresses Tat-SEC interaction. JQ1 antagonizes this effect of BRD4 by displacing it from chromatin (disrupted BRD4-Ac-histone interaction) and promoting the formation of a functional Tat-SEC (CDK9/Cyclin T1/ELL2). Tat-SEC leads to RNAPII phosphorylation and productive HIV transcriptional elongation (based on Li et al. [25]). **b** BRD4 inhibition by ZL0580. ZL0580 induces a distinct effect from JQ1. By targeting the non-KAc site on BRD4, ZL0580 does not disrupt BRD4-Ac-histone interaction (no displacement of BRD4 from chromatin) but promotes BRD4 interaction with p-TEFb/CDK9, leading to competitive reduction in Tat binding to p-TEFb/CDK9. In addition, ZL0580 treatment also down-regulates ELL2 protein by reducing its protein stability. Reduced Tat-CDK9 binding and down-regulated ELL2 together lead to reduced RNAP phosphorylation at the HIV LTR and abortive HIV transcription elongation

**Fig. 3:**
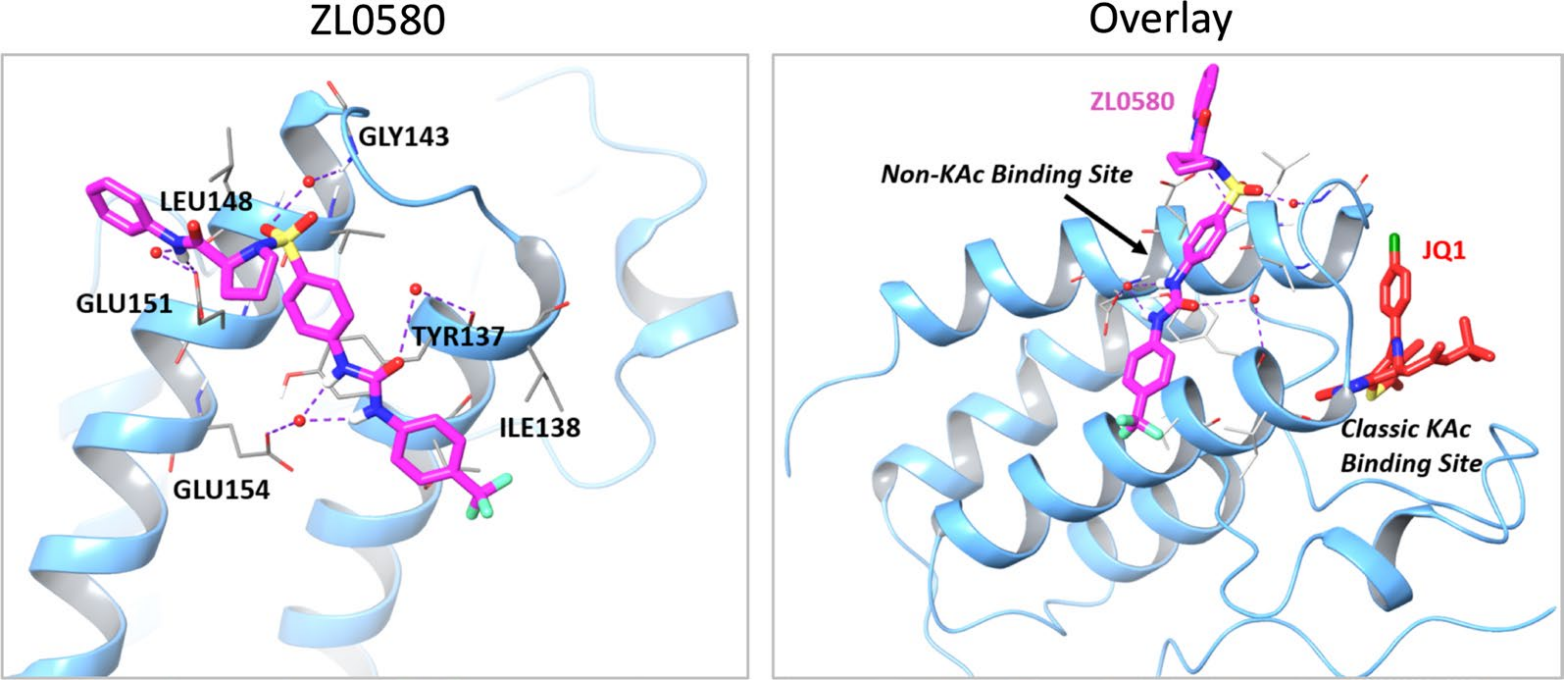
Docking analysis. Ribbon representation of ZL0580 (magenta stick) docked into BRD4 BD1. ZL0580 can be well docked into a distinct new non-KAc binding site in a similar composition. (Right) Overlay analysis of binding poses of ZL0580 (in magenta) at a distinct new non-KAc protein–protein interaction recognition site in comparison with JQ1 (in red) at the classic KAc binding site of BRD4 BD1

Our recent study [43] identified that ZL0580 inhibits Tat transactivation in J-Lat cells by decreasing its binding to CDK9 and reducing its recruitment to the HIV LTR. By contrast, JQ1 induces an opposing effect from ZL0580 and enhances CDK9 binding to Tat [43], which is consistent with a previous study reporting that JQ1 promotes Tat transactivation by relieving the inhibitory effect of BRD4 on Tat-CDK9 binding [25]. Our study further showed that ZL0580 and JQ1 also induce opposing profiles of CDK9-BRD4 binding: JQ1 decreases CDK9 binding to BRD4, whereas ZL0580 enhances CDK9 binding to BRD4 [43]. Similar effects on Tat transactivation by ZL0580 compared with JQ1 was also observed in myeloid/microglial cells [44]. These findings indicate that ZL0580 and JQ1 differentially regulate the PPI network of BRD4, leading to their distinct effects on HIV transcription. These findings on the distinct effects of ZL0580 and JQ1 on BRD4 PPI and Tat-CDK9 binding is summarized in a schematic model (Fig. 2). In the absence of compound treatment, BRD4 binds to chromatin Ac-histones (via the KAc binding pocket on its BDs) and recruits cellular p-TEFb/CDK9 via its p-TEFb-interacting domain (PID), thereby competitively limiting CDK9-Tat binding. JQ1 treatment displaces BRD4 from Ac-histones and also releases CDK9 from BRD4, thereby promoting Tat-CDK9 binding [25] (Fig. 2a). In contrast, ZL0580 treatment does not dissociate BRD4 from Ac-histones since it does not disrupt the BRD4 KAc binding pocket (to be discussed in Fig. 3). Instead, ZL0580 promotes BRD4 binding to CDK9, which competitively inhibits Tat-CDK9 binding (Fig. 2b). In support of these findings, our ongoing study employing RNA-Seq to characterize the transcriptomic profile of cells treated with each individual compound reveals a consistent pattern that ZL0580 induces an opposing transcriptomic profile to JQ1 (data not shown). Molecular mechanisms for how ZL0580 and JQ1 regulate the BRD4 PPI and thereby distinctly regulate HIV and cellular gene expression, remain unclear and warrant further investigation.

Formation of a functional super elongation complex (SEC), consisting of p-TEFb/CDK9, ELL2 and other proteins, is critical for productive HIV transcription [71]. Similar to CDK9, ELL2 is another catalytic unit in SEC to stimulate RNAP-II activation [72]. Our recent study made an intriguing observation that ZL0580 and JQ1 engage BRD4 to distinctly regulate the ELL2 protein [43]: ZL0580 down-regulates ELL2 by reducing its protein stability in cells, whereas JQ1 up-regulates ELL2 expression. Such regulatory effects of ZL0580 compared with JQ1 on ELL2 are mediated through BRD4 [43]. These findings on the distinct effects of ZL0580 and JQ1 on ELL2 regulation is summarized in Fig. 2. These data suggest that selective downregulation of ELL2 and SEC assembly may represent a potential new mechanism by which ZL0580 engages BRD4 to suppress HIV transcription. The molecular mechanisms for how ZL0580 engages BRD4 to destabilize ELL2 protein and how ZL0580/BRD4-induced ELL2 deficiency confers HIV transcriptional suppression warrants further investigation in future studies.

### Induction of repressive chromatin structure in the HIV LTR by ZL0580

Transcription from HIV provirus is driven by the 5′-LTR (long terminal repeats), which serves as a transcriptional promoter for HIV. During latent state, 2 nucleosomes (Nuc-0, and Nuc-1) are formed within the HIV promoter. Positioning of Nuc-1 downstream of the HIV TSS (transcription starting site) effectively restricts HIV transcription [73]. Nuc-1 is subjected to epigenetic modifications that change the accessibility of DNA and therefore contributes to HIV silencing [73, 74]. Nucleosomal structure can be epigenetically modified through chromatin remodeling proteins (e.g. BAF/PBAF), which can restructure nucleosome to alter histone affinity for DNA [73, 74]. A recent study identified that BRD4 can engage the cellular chromatin remodeling protein, BAF, to alter nucleosome structures at HIV LTRs, inducing a repressive HIV LTR structure [23]. BRD4 recruits BAF [23], which mediates positioning Nuc-1 downstream of the TSS, inducing HIV transcriptional repression [73]. Our studies [43, 44] showed that other than in activated cells, our BRD4 modulator ZL0580 could also suppress basal HIV transcription in resting cells where Tat protein levels are low, indicating that ZL0580 may also suppress HIV transcription via mechanisms independent of Tat or transcription elongation (such as CDK9, ELL2, RNAP-II, and SEC). Notably, our study also found that ZL0580 could durably repress latent HIV from reactivation in multiple cell models, including J-Lat cells, patient PBMCs [43], and microglia [44]. Based on these data and the role of BRD4 in regulating nucleosomal structure, we speculate that ZL0580 may induce epigenetic reprogramming of the HIV LTR. Our study explored the epigenetic profile of the HIV promoter after treatment with ZL0580 as compared to JQ1 using high-resolution MNase nucleosomal mapping [73]. The data showed that ZL0580 induces more repressive chromatin structure at the HIV LTR by enhancing nucleosomal DNA protection in the majority of the amplicon regions of the HIV LTR, especially in amplicon 13, which covers Nuc-1 immediately downstream of the TSS [43]. Similar results were also observed in microglia [44]. Therefore, our studies indicate that ZL0580 engages BRD4 to enhance heterochromatinization of the HIV promoter to induce deeper latency. The molecular mechanisms for how ZL0580 induces repressive HIV LTR structure through BRD4 remain less clear but may involve ZL0580-induced BRD4 engagement of chromatin modifiers and/or remodeling proteins (Fig. 1) [23, 73, 75]. Identification of novel BRD4-interacting proteins following ZL0580 treatment compared to JQ1 treatment is considered critical and should provide new insights into the mechanisms of action for these two molecules as well as into our understanding of the basic biology of BRD4 in HIV epigenetic regulation.

## Molecular basis for distinct binding modes of ZL0580 and JQ1 to BRD4/BET

Targeted modulation of a protein or pathway by different regulatory agents (e.g. agonist and antagonist) to induce distinct functional outcomes has been reported. Our studies have presented multiple lines of evidence supporting the targeting of BRD4 by ZL0580 to induce HIV epigenetic suppression [43]. In an attempt to understand why modulation of the same protein by ZL0580 or by JQ1 could induce distinct functional outcomes of HIV transcription, our study explored the structural basis for binding of ZL0580 and JQ1 to BRD4. In vitro binding assays (time-resolved fluorescence energy transfer) showed that, different from JQ1 which non-selectively binds to both BD1 and BD2 of all BET proteins as a pan-BET inhibitor [33], ZL0580 selectively binds to the BRD4 BD1 domain (IC50 = 163 nM) [43]. Our study also explored recognition sites of BRD4 by ZL0580 compared with JQ1 using a docking analysis based on the already determined BRD4/BD1 co-crystal structure [33]. As shown in Fig. 3, our analysis indicated that ZL0580 can be docked into a new non-acetyl-lysine (KAc) binding site located at the helix αB and αC surface containing BRD4 key residues (e.g., Glu151). ZL0580 can form strong and critical interactions with Glu151, Glu154, Tyr137, Gly143 and Leu148 residues via H-bonds (purple dotted line) (Fig. 3) (left). An overlay analysis of binding poses of ZL0580 revealed a distinct non-KAc binding site of BRD4 BD1 in comparison with JQ1 at the classic KAc binding site that is at the end of four helix bundles (Fig. 3) (right). Therefore, the docking analysis indicates a unique recognition site for ZL0580 on BRD4 (possibly involved in protein–protein interactions) with notable differences from that of JQ1. A detailed co-crystal analysis needs to be conducted in the future to confirm the structural basis for the distinct binding mode of ZL0580 to BRD4 compared to that of JQ1.

## Conclusion

Recent studies by our group and others indicate that the biology of BRD4 in the context of HIV epigenetic regulation is more complex than currently anticipated and that BRD4 protein and its associated host epigenetic machinery could be modulated to induce HIV suppression, paving a potential new way for “block and lock” HIV therapy. Several critical questions remain to be investigated. First, although our ongoing studies have chemically optimized the lead candidate ZL0580 and have identified additional more potent analogs, we will continue our lead optimization, and it is hoped that through comprehensive optimization, we can further improve the potency and the durability of this class of molecules in suppressing HIV. In addition, it is important that these key findings will be independently confirmed by additional studies from other groups. Second, further research is needed to understand in more detail how such BRD4 modulators induce HIV repression. For example, such mechanisms may include, but are not limited to, the recruitment of repressive chromatin remodeling proteins, BRD4-mediated protein–protein interactions, and other downstream pathways involved in ZL0580-induced HIV suppression. This research will not only improve our understanding of the mechanisms of action for ZL0580, but will also provide new insights into the basic biology of HIV proviral regulation and latency. Third, protein–ligand co-crystal analyses are needed to understand the structural basis for distinct interactions of this class of molecules with BRD4 from other BET inhibitors such as JQ1. Finally, future in vivo studies such as the evaluation of pharmacological and toxicological properties of this class of compounds including DMPK (bioavailability, half-life, clearance and metabolic profile), as well as in vivo efficacy to repress latent HIV either alone or in combination with other HIV silencers in animal models, are warranted to better evaluate the potential utility of this class of molecules in HIV epigenetic suppression and silencing.

## Data Availability

All data are included in this article.

## Abbreviations

HIV: Human immunodeficiency virus;
BRD4: Bromodomain (BD) and Extra-Terminal Domain (ET) protein 4
BET: Bromodomain and extra-terminal domain proteins
ART: Antiretroviral therapy
KAc: Acetyl-lysine
LTR: Long-terminal repeats
AcH3: Acetylated histone 3
AcH4: Acetylated histone 4
RNAPII: RNA polymerase II
p-TEFb: Positive transcription elongation factor
CDK9: Cyclin-dependent kinase 9
TR-FRET: Time-resolved fluorescence energy transfer
PMA: Phorbol 12-myristate 13-acetate
CNS: Central nervous system
LRA: Latency reversing agents
TAR: Transactivation-responsive element
dCA: Didehydro-cortistatin A
PPI: Protein–protein interaction
SEC: Super elongation complex
ELL2: Elongation factor For RNA polymerase II 2
TSS: Transcription start site
PBMC: Peripheral blood mononuclear cells
SWI/SNF: SWItch/sucrose non-fermentable
